# Protein Delivery Using Three-Dimensional Printing of Buccal Films: Technological Advances and Clinical Potential

**DOI:** 10.3390/pharmaceutics18070789

**Published:** 2026-06-27

**Authors:** Tejaswi Appidi, Thirupathi R. Anekalla, Shanthi Chede, Leela Raghava Jaidev Chakka, Mohammed Maniruzzaman

**Affiliations:** 1 Department of Pharmaceutics and Drug Delivery, School of Pharmacy, University of Mississippi, Oxford, MS 38677, USA; tappidi@go.olemiss.edu (T.A.); tanekall@go.olemiss.edu (T.R.A.); 2 Department of Pharmaceutics, School of Pharmacy, University of Iowa, Iowa City, IA 52242, USA; santimonist@gmail.com

**Keywords:** protein drug delivery, buccal films, non-invasive drug delivery, 3D printing, personalized medicine

## Abstract

Therapeutic proteins have emerged as a cornerstone of modern medicine due to their high specificity and strong biological effects. However, delivering these proteins poses significant challenges due to their instability, susceptibility to enzymatic breakdown, low permeability, and reliance on invasive parenteral routes. Buccal drug delivery is a promising non-invasive alternative, offering quick systemic absorption while avoiding gastrointestinal degradation and hepatic first-pass metabolism. Three-dimensional (3D) printing as a fabrication method has further enhanced the potential of buccal delivery, enabling precise dosage control, multilayer structures, and patient-specific customization. This review focuses on the current state of the traditional and 3D-printed buccal film platforms using different printing methods for protein delivery, and critically analyzes protein stability challenges, and formulation strategies. The discussion further highlights emerging proof-of-concept studies.

## 1. Introduction

**Figure 1:**
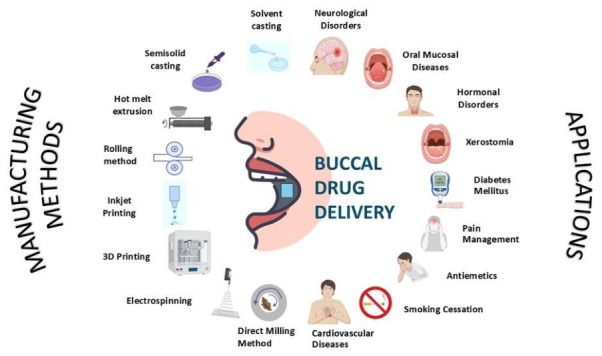
Overview of buccal drug delivery—manufacturing methods and applications. Created in BioRender. Appidi, T. (2026) https://BioRender.com/nubwphh (accessed on 23 June 2026).

Proteins are biologically abundant macromolecules composed of amino acids linked by peptide bonds and capable of adopting structures ranging from simple linear chains to highly complex three-dimensional conformations [1]. Therapeutic proteins, including hormones (like insulin and growth hormone), monoclonal antibodies, cytokines and interferons, enzymes used in replacement therapy, growth factors, fusion proteins, blood factors, and protein-based vaccines, have transformed modern pharmacotherapy because of their high specificity, strong biological activity, and relatively low off-target toxicity [2,3]. Conventionally, protein-based therapeutics are delivered through the parenteral route due to the numerous physiological and formulation challenges that hinder effective oral administration [3,4]. However, repeated doses/injections are associated with pain, infection risk, increased healthcare burden, and compromise on drug adherence at the site to elicit a therapeutic response [3,5,6]. The drug delivery to the buccal mucosa has attracted increasing attention over the past two decades as an attractive site for the delivery of macromolecules [7]. Buccal delivery offers a promising non-invasive route for systemic administration of macromolecules, bypassing gastrointestinal and hepatic first-pass metabolism, and is suitable for a range of therapeutic applications (Figure 1). It provides several advantages, such as relative physical robustness, easy accessibility, and the potential for rapid onset along with improved patient adherence [7,8]. However, the delivery remains challenging due to limited permeability, dynamic salivary environment, continuous salivary washout, restrictions on drug loading, and the inherent fragility of proteins, resulting in low bioavailability and complex formulation requirements [3,7]. To overcome these barriers, strategies like mucoadhesive polymers, permeation enhancers, enzyme inhibitors, and nanocarriers have been investigated to protect proteins and enhance mucosal transport [3,6,7].

Conventional manufacturing of buccal films by solvent casting or hot-melt extrusion offers limited flexibility for dose customization and internal structure [7,9]. In contrast, three-dimensional (3D) printing technologies allow precise control over film geometry, spatial distribution of APIs and excipients, multilayer or compartmentalized designs, and on-demand, patient-specific doses (Figure 1, Table 1) [7,10]. Importantly, semi-solid extrusion printing can be performed under mild processing conditions, making it particularly suitable for protein and peptide delivery by minimizing degradation and preserving biological activity. This review highlights technological progress and the clinical potential of 3D-printed buccal films for protein delivery, focusing on formulation approaches, 3D printing techniques, and related proof-of-concept studies.

## 2. Therapeutic Proteins: Opportunities and Delivery Challenges

### 2.1. Role of Proteins in Modern Therapy

**Figure 2:**
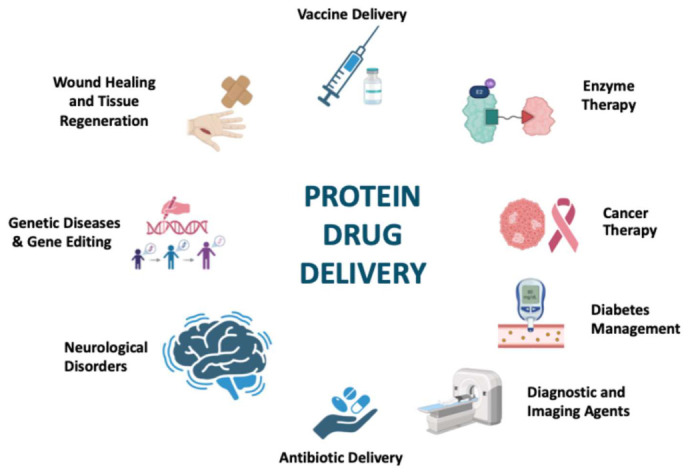
Diagram showing the versatility of protein delivery for different disease conditions. Created in BioRender. Appidi, T. (2026) https://BioRender.com/amrgv5l (accessed on 23 June 2026).

Therapeutic proteins are among the fastest growing drug categories, with hundreds of approved products and an annual market nearing $400 billion [3,13]. These molecules are essential clinically, in treating diabetes, cancer, hormonal, genetic, and metabolic disorders, autoimmune and inflammatory conditions, cardiovascular issues, and infectious diseases, and are also fundamental to modern vaccinology (Figure 2) [14]. Compared to small molecules, proteins generally exhibit higher target specificity and stronger efficacy, with reduced off-target toxicity. This enables precise control of signaling pathways and the ability to substitute for missing or defective proteins [3,13]. Their structural complexity supports functions that are difficult or impossible to replicate with traditional chemotypes, which involve complex receptor interactions, catalytic activity in enzyme replacement, or multivalent immunological responses [14,15]. The versatility of the proteins has driven rapid expansion in areas like bispecific antibodies, fusion proteins, and conditionally active cytokines [14]. The key advancements in recombinant DNA technology, protein engineering, and computational protein design have expanded the range and effectiveness of protein-oriented therapies [13]. Modern techniques for design that include structure-based modeling, machine learning, and directed evolution are producing new scaffolds as well as enhanced antibodies, enzymes, and cytokines with better pharmacokinetics and manufacturability [13,14,15].

### 2.2. Challenges and Formulation Strategies in Protein Delivery

Therapeutic proteins are inherently fragile macromolecules that are poorly permeable and have narrow stability windows [16,17]. Despite advantages and varied therapeutic opportunities to explore, many therapeutic proteins face issues [18,19].

#### 2.2.1. Protein Stability—pH, Environment, and Mechanical Factors

A major challenge in delivering proteins is maintaining their structural and conformational stability. Protein conformation is maintained by a delicate balance of electrostatic interactions, hydrogen bonds, van der Waals forces, and hydrophobic effects; any disruption can cause unfolding, aggregation, and a loss of biological activity [16,20,21]. In this regard, pH plays a crucial role by influencing amino acid ionization, intramolecular electrostatics, and colloidal stability; even slight deviations from optimal conditions can lead to denaturation, deamidation or aggregation [22,23]. Similarly, temperature variations outside the protein’s stability range can also induce thermal denaturation, oxidation, hydrolysis, and precipitation, while repeated free-thaw cycles or inadequate cold-chain management further compromise stability of the products [16,24]. Furthermore, exposure to light and reactive oxygen species can oxidize vulnerable residues, leading to structural alterations and aggregation [25]. In addition, proteins are also highly affected by mechanical and interfacial stresses during upstream processing, including pumping, filtration, filling, transport, and administration [25,26]. Shear forces and adsorption at air-liquid or solid–liquid interfaces can cause partial unfolding and aggregation. To counteract these effects, formulations utilize optimized buffers, pH and ionic strength adjustments, sugars and polyols, amino acids, surfactants, antioxidants, and gentle processing conditions, but maintaining stability across the full product lifecycle remains challenging [27].

#### 2.2.2. Solubility, Permeability, and Immunogenicity Problems

In addition to instability issues, limitations in solubility and permeability further challenge effective protein delivery. Many proteins exhibit poor solubility near physiological pH and tend to self-aggregate at high concentrations, which restricts dose loading and promotes aggregation [16,28,29]. Their large size and hydrophilic nature greatly hinder passive membrane diffusion, resulting in minimal uptake across the cellular and epithelial layers without specialized transport mechanisms or permeation enhancers [30]. In particular, the oral route of delivery poses the greatest challenges, as proteins must withstand extreme pH changes and gastric acidity while being exposed to proteases that denature and degrade them into inactive fragments [30]. Despite some absorption, extensive first-pass hepatic metabolism significantly reduces systemic exposure, resulting in bioavailability often dropping below 1% for unprotected proteins [22]. Lipid-based systems, nanoparticles, enteric coatings, enzyme inhibitors, and absorption enhancers can improve solubility, protection, and permeability to some extent, However, achieving consistent efficacy in humans remains challenging [31]. A major obstacle in developing therapeutic proteins is immunogenicity. Factors such as structural instability, chemical modifications, and aggregation increase the risk of anti-drug antibody (ADA) formation, which can neutralize the drug, alter its pharmacokinetics, or cause hypersensitivity and other adverse immune responses [32]. The immunogenic risk is also affected by the administration route, such as subcutaneous depots with prolonged antigen exposure, and formulation aspects like impurities, particulates, and excipient choices. These issues collectively complicate long-term therapy, often requiring dose escalation, changes in dosing schedules, or switching to different biologics [29,32,33].

#### 2.2.3. Formulation Approaches to Address Protein Delivery Issues

To address difficulties in protein delivery, various molecular, formulation, and route specific techniques have been explored. Molecular strategies like PEGylation, lipidation, glycosylation, cyclization, Fc-fusion, and albumin binding enhance hydrodynamic size, stability, and circulation half-life, while reducing proteolysis [34,35,36,37]. Formulation techniques employ hydrogels and nanocarriers (polymeric nanoparticles, lipid-based systems, micelles, liposomes and microspheres) to protect proteins from harsh conditions, enable controlled release, and increase absorption, particularly for oral delivery [22,38]. For gastrointestinal delivery, strategies such as enteric coatings, enzyme inhibitors, permeation enhancers, mucus penetrating or mucoadhesive systems, intestinal microdevices, and cell-targeted carriers are designed to bypass enzymatic and gastric degradation and overcome epithelial barriers [6,22]. In parallel, non-invasive methods like oral, pulmonary, nasal, transdermal, buccal, ocular, and rectal are explored to improve adherence and enable local or systemic delivery without injections, though each has distinct anatomical and physiological constraints. Among these mucosal routes, buccal delivery is particularly promising and potential route because of its therapeutic advantages and high patient acceptability, thereby providing a natural transition to more specialized platforms, such as 3D-printed protein dosage forms, where geometry, layering, and micro-architecture can be precisely tailored to overcome the stability and transport barriers discussed earlier [6].

## 3. Buccal as a Route of Delivery

Buccal drug delivery involves placing a dosage form against the inner cheek, allowing the drug to be absorbed through the buccal mucosa to produce local or systemic effects [39,40]. This route is especially useful for drugs that experience significant first-pass metabolism or are unstable in the gastrointestinal tract, since absorption through the buccal vasculature provides direct access to systemic circulation and bypasses gastric acid and liver metabolism [7]. The buccal mucosa is made up of non-keratinized stratified squamous epithelium, measuring 500–800 µm, positioned over a highly vascular lamina propria. It provides a neutral pH, low enzymatic activity, and quick cellular recovery, all of which support drug stability and enhance mucosal tolerability [41]. Despite its limited surface area of 50 cm^2^ and the potential for saliva to dilute or remove formulations, the tissue’s moderate permeability, durability, and accessibility make it suitable for mucoadhesive, retentive systems like films, patches, and tablets [42]. Drug transport takes place through transcellular pathways that favor lipophilic molecules and paracellular routes that allow small hydrophilic molecules but are limited by tight junctions. Therefore, permeability strongly depends on molecular size and lipophilicity [43].

### 3.1. Key Parameters in Buccal Drug Delivery Systems (BDDS)

Critical parameters influencing buccal drug delivery systems (BDDS) encompass drug properties, formulation techniques, physiological factors, technological approaches, and patient-specific considerations and are particularly important for the successful administration of proteins and other biologics. While low molecular weight (<500 Da) favors passive buccal absorption, therapeutic peptides and proteins exceed this limit and therefore require formulation strategies like permeation enhancers, enzyme inhibitors, and mucoadhesive systems to overcome epithelial barriers and reach therapeutic levels [44,45]. Optimal candidates show moderate lipophilicity along with high aqueous solubility, which helps the drug’s diffusion across the mucosal barrier while maintaining adequate drug availability at the absorption site [9]. Due to the limited surface area of the buccal mucosa, BDDS are ideal for potent drugs that need low doses [46]. Additionally, the active pharmaceutical ingredient must stay stable in the enzymatically active, moist environment of saliva during its residence time [44,47]. Mucoadhesive polymers, both natural, like chitosan, gelatin, and alginate, and synthetic, such as HPMC (hydroxypropyl methylcellulose), Carbopol, and PVA (polyvinyl alcohol), are essential for extending mucosal contact and facilitating controlled drug release [9,45]. The addition of permeation enhancers, enzyme inhibitors, pH modifiers (targeting an optimal pH of 6.5–7.5), film-formers, and appropriate plasticizers, such as PEG and glycerol, further improves the drug’s solubility, permeability, and the mechanical properties of the dosage form [9,44,45]. Designing the dosage form should focus on comfort, ease of application, and effective mucoadhesion, typically using systems like mucoadhesive films, buccal tablets, patches, and hydrogels [9,47].

Physiological factors like mucosal thickness, saliva flow, and local enzymatic activity affect drug retention, dissolution, and metabolism. Therefore, designing formulations that are resistant to salivary washout and enzymatic degradation is crucial, especially for biologics [9,44,45,46]. Technological and manufacturing factors, such as advanced methods like semi-solid extrusion-based 3D printing, facilitate the creation of precise, customizable, and multilayered buccal systems. However, ensuring stability, sterility, scalability, and reproducibility across batches is essential for clinical use and commercialization [9,44,45,46,47]. Finally, patient-centered and regulatory considerations like palatability, comfort, discreet and user-friendly design, as well as strict adherence to biocompatibility, safety, and efficacy standards are essential for ensuring patient adherence and regulatory approval, particularly for innovative and personalized buccal drug delivery systems [9,44,45,46].

### 3.2. Buccal Permeation Enhancers

The buccal mucosa serves as a significant barrier, especially for hydrophilic and macromolecular drugs such as proteins. Buccal permeation enhancers (BPEs) can temporarily alter epithelial barriers to boost drug absorption. The buccal route provides a favorable alternative to both parenteral and oral delivery because of its multiple therapeutic advantages [48]. However, the main obstacle is the physicochemical barrier of the buccal epithelium, which is a stratified squamous non-keratinized structure with tight intercellular lipid zones. To overcome this, various BPEs have been created to enhance drug flux across the mucosa. These agents act through mechanisms such as membrane fluidization, modulation of tight junctions, mucoadhesion, and chelation. In recent years, there has been notable progress in the design, characterization, and application of next-generation BPEs. These advancements aim to increase permeability while maintaining safety, reversibility, and compatibility with innovative drug delivery systems.

One of the significant recent breakthroughs is the use of biophysical characterization tools, such as electron spin resonance (ESR), as demonstrated by Chede et al. (2021), who measured the fluidizing effects of sodium caprylate and l-menthol on model buccal membranes [49]. These tools provide predictive insights into enhancer potency, enabling rational selection based on lipid bilayer interactions rather than empirical screening. Functionalized polymers, such as N-trimethyl chitosan, thiolated chitosan, and Carbopol derivatives, have been developed to enhance both mucoadhesion and permeation. These polymers modulate tight junctions and exhibit site-specific bioadhesion, resulting in enhanced paracellular and transcellular drug transport with minimal irritation [50]. Combinations of enhancers such as chitosan with fatty acids or bile salts with essential oils have demonstrated synergistic effects, enhanced drug flux while allowing for lower concentrations of each component. This decreases potential toxicity while maintaining effectiveness. The emergence of stimuli-sensitive materials such as pH-sensitive or thermo responsive enhancers enables drug release and permeation to be triggered by changes in the buccal cavity, enhancing localized effects and reducing systemic exposure. These materials are particularly beneficial for buccal films and hydrogels that require on-demand activation. Lipid nanoparticles, nanoemulsions, and polymeric micelles with permeation enhancers improve solubility and increase the permeation of poorly absorbed molecules. These systems keep the enhancer and drug close together, boosting epithelial uptake while reducing enhancer diffusion away from the site. There is growing interest in Generally Recognized As Safe (GRAS) agents such as essential oils (e.g., eugenol, thymol), l-menthol, and citric acid, which offer acceptable safety profiles for chronic or repeated buccal use. These are especially beneficial in over-the-counter therapeutics and nutraceuticals. Specialized BPEs are now being developed for the delivery of macromolecules. Sodium caprylate, L-menthol, and bile salts have been shown to enhance the buccal delivery of peptides (e.g., insulin, growth hormone) by temporarily altering lipid fluidity without causing permanent membrane damage [49,50].

## 4. Conventional Buccal Film Platforms for Protein Delivery

Conventional buccal films for proteins and peptides are thin, mucoadhesive polymer matrices placed on the inner cheek to deliver the drug across the oral mucosa (Table 2) [7,51]. The dominant manufacturing method is solvent casting, in which polymers and excipients are dissolved or dispersed with the protein, cast as a uniform solution, and dried into flexible films. This approach is widely adopted industrially because it is simple, scalabe, and compatible with sensitive biologics [52,53]. Conventional hot melt extrusion has also been used, but thermal stress can limit its application to proteins [54]. To improve protein stabilization and transport, nanoparticles in film systems are increasingly being incorporated into conventional buccal films. Protein-loaded polymeric or lipid nanoparticles (often chitosan-based, PEGylated, or deformable vesicles) are prepared separately and then embedded within a mucoadhesive film matrix, improving enzymatic protection, local retention, and buccal permeation. Such hybrid systems have been particularly explored for insulin, where buccal films embedding chitosan or phospholipid nanoparticles reduced blood glucose in diabetic animals and demonstrated significant mucosal permeation in vitro [7,54].

Cellulose-ether-based mucoadhesive films are highlighted as especially suitable for biological products like proteins and peptides because they can be tailored as fast dissolving or slowly eroding systems, provide strong adhesion and unidirectional release, and can be manufactured by standard techniques such as solvent casting, inkjet/3D printing, and electrospinning [51]. Despite these advances, translation to marketed protein-loaded buccal films remains limited; the most advanced example, a dissolvable insulin film containing gold glycan-coated nanoparticles (Pharma Film), reached clinical trials but failed due to low buccal bioavailability, underscoring that conventional platforms still struggle with drug loading and achieving therapeutically relevant systemic exposure for large hydrophilic proteins [7,8,54].

## 5. Fabrication of Buccal Films Using Three-Dimensional (3D) Printing

**Figure 3:**
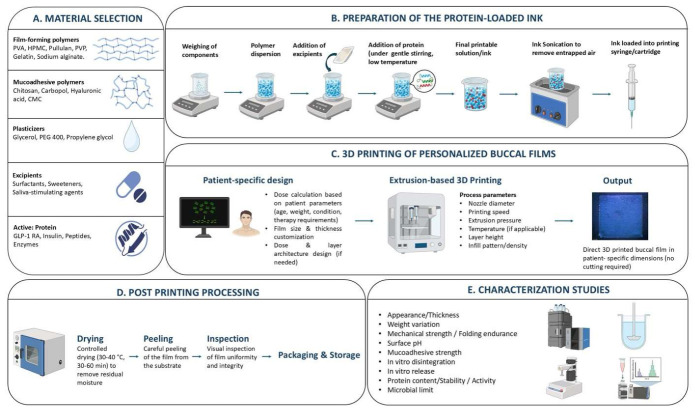
Image illustrating the typical process of selecting materials, manufacturing, and 3D printing process for preparing protein-loaded buccal films with a tailored dose for drug release applications. Created in BioRender. Appidi, T. (2026) https://BioRender.com/n0isl6o (accessed on 23 June 2026).

Three-dimensional (3D) printing has become a flexible method for creating personalized, complex buccal films (Figure 3). It overcomes the major drawbacks of traditional solvent casting, including limited dose flexibility, residual solvents, uneven thickness, dose inconsistency, drug recrystallization, and limited geometric options control [61,62]. However, fabricating protein-loaded buccal films with 3D printing demands careful attention to formulation composition, material properties, printing parameters, and post-processing conditions to ensure both the film’s effectiveness and protein stability. Unlike small-molecule drugs, proteins are very sensitive to factors such as temperature, pH, shear forces, and dehydration, which require tailored manufacturing strategies.

### 5.1. Materials Selection and Manufacturing Processes of Protein-Loaded Buccal Films

#### 5.1.1. Formulation Development and Material Selection

The initial stage involves choosing film-forming and mucoadhesive polymers that enable printable formulations without compromising protein integrity. Common polymers include hydroxypropyl methylcellulose (HPMC), polyvinyl alcohol (PVA), hydroxypropyl cellulose (HPC), chitosan, sodium alginate, and Carbopol. These polymers ensure adequate mechanical strength, flexibility, and adhesion to the buccal mucosa. Plasticizers such as glycerol and polyethylene glycol (PEG) are added to enhance film flexibility and reduce brittleness. Protein stabilizers, including trehalose, sucrose, mannitol, and amino acids, are often included to protect protein structure during manufacturing and storage. Additional excipients, such as permeation enhancers, buffering agents, and viscosity modifiers, may also be included depending on the desired therapeutic outcome [63,64].

#### 5.1.2. Hydrogel Ink Preparation for Semi-Solid Extrusion Printing

Semi-solid extrusion (SSE) is the most widely used method for 3D-printing protein-loaded buccal films due to its gentle processing conditions. This technique involves gradually dispersing polymers in purified water with continuous stirring until they are fully hydrated. Subsequently, plasticizers and stabilizing excipients are added to form a uniform hydrogel matrix. The protein is incorporated during the final formulation step under controlled mixing to minimize shear-induced denaturation. The resulting hydrogel is then degassed to remove entrapped air bubbles that could compromise printing accuracy and film uniformity. The rheological properties of the hydrogel are critical for successful printing. Specifically, the formulation should exhibit shear-thinning behavior, enabling smooth extrusion through the nozzle while maintaining sufficient viscosity and yield stress to preserve the printed structure after deposition [12,65].

#### 5.1.3. Semi-Solid Extrusion Printing Process

During SSE printing, the hydrogel ink is loaded into a syringe-based extrusion system and deposited layer by layer onto a printing platform based on a computer-aided design (CAD) model. The process typically occurs at room temperature, minimizing thermal degradation of proteins. Various process parameters influence print quality and dosage uniformity, including nozzle diameter, layer height, printing temperature, extrusion pressure, printing speed, infill density, and printing pattern. Optimizing these parameters is essential to achieve uniform film thickness, reproducible drug loading, and precise geometric dimensions. Furthermore, excessive pressure or prolonged residence time within the syringe should be avoided to minimize protein exposure to mechanical stress [12,66].

#### 5.1.4. Post-Printing Processing

Following printing, buccal films undergo drying or conditioning to remove excess moisture and enhance mechanical stability. Air drying at controlled temperature and humidity is commonly employed. For highly sensitive proteins, lyophilization may be used to preserve biological activity and minimize structural changes. The drying process must be carefully controlled, as rapid moisture loss can result in film cracking, shrinkage, or protein aggregation. Residual moisture content also plays a significant role in long-term protein stability [67].

#### 5.1.5. Quality Control and Characterization

Manufactured films undergo extensive characterization to evaluate their physicochemical properties and therapeutic performance. Key quality attributes include film thickness and weight uniformity, surface morphology, drug content uniformity, in vitro release behavior, mechanical strength and flexibility, mucoadhesive properties, and buccal permeation performance [68]. For protein-containing formulations, additional analytical techniques such as circular dichroism spectroscopy, differential scanning calorimetry, Fourier-transform infrared spectroscopy, enzyme-linked immunosorbent assays, and high-performance liquid chromatography are often employed to confirm the protein’s structural integrity and biological activity after printing [69].

#### 5.1.6. Manufacturing Challenges

Three-dimensional printing of buccal films for protein delivery is currently limited to a narrow set of polymers that can be printed reliably while still providing muco-adhesion, mechanical strength, and biocompatibility. Reviews of oral and buccal films highlight that only a few film-forming polymers, such as PVA, PVP, gelatin, and selected cellulose derivatives, are routinely used in 3D-printed oral systems, and these often need plasticizers or blending to achieve suitable flexibility and strength [12]. Many established mucoadhesive polymers identified in buccal film research lack either adequate processability in extrusion or inkjet-based printing, or sufficient mechanical integrity when printed alone [70]. For biologics, buccal films have mainly been made by solvent casting, with newer methods such as hot-melt extrusion, FDM, and inkjet printing only recently explored; thermal and shear stresses in FDM/HME risk degrading heat- sensitive drugs, while solvent-based processes raise concerns about protein stability and residues [8,71]. Additionally, extrusion-based printing demands a tight rheological window with appropriate viscosity, viscoelasticity, and yield stress for accurate layer deposition and shape fidelity, and this balance can be disrupted by protein loading. Together, these factors mean that truly protein-friendly, printable, and strongly mucoadhesive buccal films can only be formulated within a limited, carefully optimized material space.

While current buccal films are mainly limited to potent drugs due to their low loading capacity per unit area. However, 3D printing allows for stacking multiple layers without increasing the film’s size, enabling higher drug content while still fitting within mucosal dimensions and ensuring mechanical comfort [7,10,51]. Compartmentalized architectures like separate layers for drugs, permeation enhancers, and taste masking, or physically isolated APIs, reduce incompatibilities and allow for effective combination therapies. Using computer-aided design, the dose, film size, and geometry can be quickly adjusted to match individual needs such as age, weight, organ function, or disease condition, and several drugs can be combined in a single film to simplify complex regimens [64]. By tuning internal infill patterns, multilayer structures, and backing layer designs, 3D-printed buccal films can be engineered for immediate, sustained, or unidirectional release, supporting tight therapeutic control [61,71]. For example, bilayer estradiol buccal films with different infill patterns showed clearly distinct release kinetics, while FDM-printed mucoadhesive films achieved unidirectional drug release using backing layers [72,73]. These design freedoms, combined with nanocarriers or smart polymers when needed, make 3D-printed buccal films a promising platform for precision medicine and personalized therapy, including challenging molecules such as cannabinoids and biologics. Together, these features, along with dense buccal vascularization and bypassing first -pass metabolism, make 3D-printed buccal films a promising platform for personalized, controlled delivery of small molecules and future protein-based therapeutics [61,74]. Table 3 presents different 3D printing methods used for buccal film preparation, highlighting their advantages, limitations, and ideal applications.

### 5.2. Three-Dimensional Printing of Model Proteins for Buccal Application—Current State of the Art

**Figure 4:**
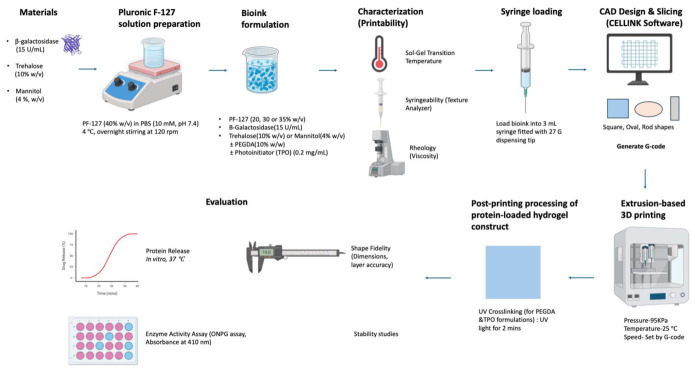
Microextrusion-based 3D printing of buccal films [98]. Created in BioRender. Appidi, T. (2026) https://BioRender.com/5ll4tj1 (accessed on 23 June 2026).

Recent progress in three-dimensional (3D) printing has enabled the fabrication of protein-based dosage forms and devices with accurate control over their structure, release profiles, and biological functions. This development positions additive manufacturing as a groundbreaking method for buccal drug delivery. Early investigations established the feasibility of thermal inkjet 3D printing for producing buccal films incorporating lysozyme and ribonuclease A. The ink formulation, consisting of a 70:30 water-to-glycerin ratio, was supplemented with sodium deoxycholate to improve permeation and was printed onto hydroxypropyl methylcellulose (HPMC) and polycaprolactone (PCL) substrates. The printed proteins preserved both their structural integrity and enzymatic activity. Additionally, the films showed favorable mucoadhesive properties, flexibility, and structural stability [96]. In 2018, the researchers expanded their study by examining the printing of lysozyme using a similar ink formulation, this time without permeation enhancers, onto HPMC/chitosan and electrospun PCL film substrates. Comprehensive analyses confirmed that the protein’s structural integrity was maintained and that the films retained their mechanical properties. These findings further support the potential of thermal inkjet printing as a gentle, reliable method for producing buccal delivery systems containing active proteins [97]. In parallel, developments in extrusion-based 3D printing have facilitated greater structural and release control in protein-loaded buccal films. Microextrusion-based 3D printing was used to develop multilayered buccal films that include β-galactosidase and ovalbumin. The formulation contained pluronic F-127, trehalose, and, optionally, polyethylene glycol diacrylate (PEGDA), with a photoinitiator to improve structural integrity. Films containing 30% pluronic and 10% trehalose retained over 82% of enzymatic activity after 4 days. The multilayer design allowed for controlled drug release, with disintegration times ranging from 5 to 20 min, and extended release up to 3 h through a backing layer. This study demonstrates the ability of extrusion-based 3D printing to maintain protein functionality while allowing precise control over drug release and film structure (Figure 4) [98].

Besides film-based platforms, cutting-edge advances in 3D bioprinting have enabled the development of double-network hydrogels composed of polyproteins and elastin-like peptides. These materials were specifically designed to replicate the biomechanical and structural features of human oral mucosa, including the buccal cavity and the hard palate. Their physiologically relevant properties make them excellent in vitro models for studying drug permeability and mucosal absorption. Additionally, the incorporation of proteinaceous components makes these hydrogels promising candidates for fundamental research in buccal drug delivery platforms [99].

Device-oriented innovations broaden the range of 3D-printed buccal protein delivery methods.

MucoJet is an innovative 3D-printed jet-injection system developed to improve protein delivery across the buccal mucosa. Made from photopolymer materials, the device uses a pressure-driven mechanism to inject antigen solutions such as fluorescein-labeled ovalbumin into mucosal tissue. In ex vivo animal models, this system significantly increased mucosal immunity, with IgG and IgA levels increasing up to a thousand-fold compared to traditional droplet methods. Although still in the preclinical stage, Mucojet is a transformative tool for non-invasive mucosal delivery of protein-based immunotherapies [100]. Similarly, a study developed DLP-printed hollow microneedle devices with built-in reservoirs designed for buccal delivery of large molecules. These microneedles were made as single, durable units that could penetrate buccal tissue safely without breaking. Ex vivo tests with porcine buccal mucosa showed a significant increase in permeability for model compounds ranging from 600 to 4000 Da. Histological analysis and TR 146 cell viability tests confirmed that the device is biocompatible and safe. Overall, this platform demonstrates strong potential for delivering therapeutic peptides and proteins systemically [101].

### 5.3. Three-Dimensional Printing of Proteins for Other Applications

Three-dimensional printing of proteins has also been studied in related pharmaceutical, biomedical, and nutraceutical fields, offering design principles that can be applied to buccal protein delivery systems (Table 4). In this Context, a bilayer film was developed using semi-solid extrusion (SSE)-based 3D printing for the delivery of estradiol, which facilitated controlled drug release and improved retention at the site of administration. Although estradiol is a steroid, this approach provides a promising framework for the targeted buccal delivery of protein analogs [72]. Beyond traditional drug delivery, 3D printing has been investigated for delivering vaccines via the buccal route. In a novel preclinical approach, the delivery of a Zika virus vaccine was studied using 3D-printed buccal dissolving films (ODFs) that embed the antigen as microparticles within a polymer matrix. Animal studies in mice showed strong immunogenic effects, with a significant increase in systemic IgG and activation of both CD4^+^ and CD8^+^ T cells. demonstrating the potential of 3D-printed buccal films as a needle-free, stable vaccine delivery platform. Although not yet tested in human trials, this technology offers promise for improving global vaccine access, especially in areas where traditional cold-chain logistics are challenging [102]. In parallel, additive manufacturing of biomedical scaffolds has highlighted the effective integration of proteins within polymeric matrices. A study aimed at advancing wound care technologies reported the fabrication of 3D-printed polylactic acid (PLA) scaffolds incorporating whey protein concentrate (WPC). This combination led to enhanced swelling capacity, accelerated biodegradability, improved cellular responses, and a controlled protein release profile. Although initially developed for wound healing, the underlying design principles, such as protein encapsulation within biodegradable matrices, are highly applicable to buccal drug delivery systems. The adaptability of this approach underscores its broader relevance in protein-based therapeutic applications [103]. Furthermore, building on advances in 3D food printing, researchers have developed bioinks based on the dual hydrophilic-hydrophobic properties of sorghum and soy-derived proteins. These formulations successfully encapsulated bioactive compounds, improving their chemical stability and enhancing bioaccessibility. While initially designed for nutritional applications, this method demonstrates clear potential for pharmaceutical translation, particularly for protecting and delivering protein-based therapeutics through buccal route. The strategy highlights the versatility of plant-based proteins in developing stable, biocompatible delivery systems [104,105].

Despite these advancements, research on fully 3D-printed buccal systems that use intact therapeutic proteins or peptides as active ingredients remains limited. Most existing protein studies involve inkjet printing of model enzymes onto film substrates, while 3D-printed buccal films and microneedles have mostly been tested with small molecules [101,106]. Critical unresolved challenges include maintaining long-term protein stability during storage in printed matrices, ensuring in vivo bioavailability and managing immunogenicity after buccal administration, and developing scalable, regulatory-compliant manufacturing methods (Table 5) [7]. However, the integration of mucoadhesive film technology, nanoparticle protection strategies, and advanced 3D printing techniques (such as FDM, SSE, inkjet, and DLP) provides a solid technological basis for developing next-generation buccal protein delivery systems.

## 6. Conclusions

Three-dimensional (3D) printing of buccal films represents a powerful platform for precise, customizable protein delivery with improved dosage control and release profiles. To advance this technology, future research should focus on identifying critical quality target product profiles (QTPPs) specific to printed protein films and developing advanced analytical methods for assessing protein integrity, release, dose uniformity, and mucosal permeation. The establishment of physiologically relevant buccal models, quantitative structure–permeability relationships, and conducting robust stability studies will be essential to ensure protein integrity. Integration of PBPK/PK modeling with in vitro data will further enhance predictions of bioavailability and support dose optimization. In parallel, successful clinical translation will depend on scalable, GMP-compliant manufacturing processes with real-time quality control and regulatory frameworks that address specific quality attributes such as dose uniformity, dimensional accuracy, and microbiological safety, alongside user-centric designs tailored for vulnerable populations, to improve acceptability and adherence. Clinically, these advancements position 3D-printed buccal films as promising platforms for delivering peptide hormones, mucosal vaccines, immunotherapies, and local oral protein therapies. Conducting well-designed first-in-human trials, supported by mechanistic PK/PD modeling, is essential for validating safety and efficacy. Strong multidisciplinary collaboration will be key for integrating these technologies into standard clinical practice.

## Figures and Tables

**Table 1:** Key advantages of 3D-printed buccal films over conventional manufacturing for protein delivery [11,12].

Parameter	Conventional Buccal Films	3D-Printed Buccal Films
Dose flexibility	Fixed-dose formulations	Personalized and adjustable dosing
Film design	Limited structural complexity	Customizable geometries and multilayer structures
Protein stability	May be affected by heat and processing conditions	Better preservation of protein integrity, particularly with semi-solid extrusion
Drug distribution	Limited control over drug placement	Precise spatial distribution of proteins within the film
Drug release	Mainly controlled by formulation composition	Tunable through both formulations and printed architecture
Personalized therapy	Limited applicability	Enables patient-specific treatment approaches
Manufacturing process	Multiple processing steps and specialized tooling	Digital, Mold-free, and rapidly adaptable fabrication
On-demand production	Not feasible	Supports decentralized and on-demand manufacturing

**Table 2:** Preclinical and Early Clinical Development of Buccal Film Platforms for Protein Delivery.

Delivery System/Dosage Form	Development Stage and Animal Model	Key Outcomes	Ref.
PharmaFilm^®^ (gold nanoparticle, Aquestive Therapeutics, Warren, NJ, USA) insulin mucoadhesive buccal film	Early clinical	It has been proven to significantly lower glucose levels in humans, but further development was halted due to low buccal bioavailability.	[55]
Albumin delivery using sodium carboxymethyl cellulose and chitosan buccal films	Preclinical (Porcine buccal mucosa-ex vivo study)	Molecular docking-guided optimization revealed that sodium carboxymethyl cellulose films exhibited higher albumin release compared to chitosan.	[56]
Buccal Chitosan mucoadhesive film with insulin-loaded PEG-b-PLA nanoparticles	Preclinical (EpiOral^TM^-ex vivo study, MatTek life sciences, Massachusetts, MA, USA)	Enhanced mucoadhesion and swelling, 70% insulin encapsulation, biphasic sustained release and improved buccal permeation.	[57]
Buccal thiolated dimethyl ethyl chitosan (DMEC-Cys) mucoadhesive films for delivery of insulin nanoparticles	Preclinical (Rabbit buccal mucosa-ex vivo study)	Enhanced insulin solubility and permeability compared to non-thiolated films.	[58]
Chitosan-based mucoadhesive insulin buccal film (MBF)	Preclinical (Sprague-Dawley albino male rats)	Films were prepared using chitosan, glycerin, and L-arginine. FTIR-MS examination of insulin MBF showed that L-arginine products insulin’s structure by interacting with chitosan, thereby decreasing the formation of disordered structures and β-strands.	[59]
Hydroxypropyl β-cyclodextrin (HPβCD)-insulin complex-loaded nanoparticles based fast disintegrating mucoadhesive film	Preclinical (male ICR mice)	Cyclodextrin complexes prevent insulin from degrading, and the nanoparticles enhance mucoadhesive properties.	[60]

**Table 3:** Three-dimensional printing methods for buccal film fabrication.

3D Printing	Advantages	Limitations	Ideal for	Ref.
Inkjet printing	High-resolution and compatible with various liquid inks, this cost-effective solution enables fast production and is ideal for personalized dosage forms.	Slow curing, limited layer thickness, and the risk of solvent interaction with previous layers.	Custom oral films with complex designs using liquid or suspension inks.	[75,76,77,78,79,80]
Fused deposition modeling (FDM)	Broad filament compatibility, strong structures, adjustable infill, and consistent layer-by-layer deposition.	Requires pre-filament preparation, risk of degrading heat-sensitive materials, and limited fine detail.	Development of durable, mechanically stable drug delivery systems.	[81,82,83,84,85,86,87]
Semisolid extrusion (SSE)	Enables room-temperature manufacturing for heat-sensitive drugs, lowering costs and reducing production steps.	Viscosity-sensitive, with a risk of nozzle clogging or dripping, limited to aqueous-based systems.	Thermolabile drug formulations in continuous SSE processes.	[87,88,89,90]
Direct powder extrusion (DPE)	Avoids high temperatures, making it ideal for fragile APIs, eliminates filament production, and is practical for clinical settings.	Risk of poor surface finish, inconsistent dosage, API degradation, and oxidation caused by pneumatic pressure.	On-site or clinical fabrication where single-step, thermal-free processing is essential.	[87]
Liquid crystal display (LCD)	Affordable setup, high resolution, safer visible-light photopolymerization, potential for new resin development.	Limited by screen lifespan, light penetration problems, unintentional resin exposure, and frequent maintenance.	Affordable, high- resolution printing with new resins for visible-light applications (e.g., dental).	[91,92,93,94,95]

**Table 4:** Three-dimensionally printed protein formulations.

Printing Method	Protein(s)	Technology	Application	Key Focus	Status	Ref.
Inkjet printing	Lysozyme, Ribonuclease-A	Thermal inkjet printing	Buccal drug delivery	High structural and enzymatic stability; used sodium deoxycholate as a permeation enhancer; gentle and efficient protein printing	Preclinical proof-of-concept; Patent WO2017120689A1 filed; no in vivo buccal absorption data yet	[96,97]
Extrusion-based printing	Whey Protein Concentrate (WPC)	3D printing with PLA scaffolds	Wound healing	Biodegradable matrix with enhanced swelling, protein-controlled release, and improved cellular response; principles adaptable for buccal delivery	Functional in vitro characterization only; clinical application pending; no buccal-specific data	[103]
Extrusion	Polyproteins, elastin-like peptides	3D-bioprinted double-network hydrogels	In vitro oral mucosa model	Biomimetic tissue like behavior; useful for permeability and absorption studies; foundational for buccal delivery research	In vitro oral mucosa models only; no direct evidence of buccal drug delivery use; aligns with the FDA Modernization Act 2.0	[99]
Extrusion	Sorghum protein, soy protein	3D food printing (bioinks)	Nutraceuticals	Enhanced chemical stability and bioaccessibility of encapsulated actives; dual hydrophilic-hydrophobic matrix for protein-based therapeutics	Demonstrated in food/nutritional printing; no direct pharmaceutical buccal delivery data yet	[104,105]
Extrusion	Zika antigen (protein microparticles)	3D-printed buccal oral films	Vaccine delivery	Strong immunogenic response (IgG, T-cell activation); stable film matrix; promising needle-free vaccine approach	Robust preclinical immune response data in mice; no human trials to date	[102]
MucoJet	Ovalbumin (Model Antigen)	3D-printed jet-injection device	Buccal mucosal vaccine/immunotherapy delivery	Achieved up to 1000× increase in immune response; pressure-driven delivery; innovative for mucosal protein immunization.	Proven efficacy in ex vivo pig and in vivo rabbit models; not yet in human trials	[100]

**Table 5:** Current capabilities and unresolved challenges in 3D-printed buccal protein delivery [10,44,61,71,74,107,108].

Key Design Aspects	What 3D Printing Enables/Solves	Key Gaps and Remaining Challenges
Structural design and dose control	Patient-specific dosing; complex/multilayer geometries; spatial separation of APIs; directional release	Scaling up complexity; dose uniformity; limited clinical evidence
Drug loading and combination therapy	Higher loading via multilayer designs; multi-drug buccal films	Printability at higher loading; API-excipient interactions; structural integrity loss
Release control	Geometry/infill-driven release; multilayer and stimulus-responsive systems	Sustained protein release; bioactivity retention; weak PK/PD correlation
Protein stability	Mild processing (inkjet/SSE/LCD); protective matrices enhance stability	Sensitivity to light/shear/oxidation; limited long-term stability data
Personalization and patient-centric approaches	On-demand patient-specific films (dose/shape/size)	Limited clinical integration; workflow complexity; lack of standardization
Manufacturing and process control	Digital design to fabrication; reproducible geometry and mass control	No mature GMP/point of care framework; material variability; limited QC tools
Regulatory and translational pathway	Existing additive manufacturing approvals (e.g., SPRITAM) support feasibility	No buccal-specific guidelines; unclear batch definition; point of care uncertainty

## Data Availability

No new data were created or analyzed in this study.

## References

[1] Chen G., Kang W., Li W., Chen S., Gao Y. (2022). Oral delivery of protein and peptide drugs: From non-specific formulation approaches to intestinal cell targeting strategies. Theranostics.

[2] Jena D., Srivastava N., Chauhan I., Verma M. (2024). Challenges and Therapeutic Approaches for the Protein Delivery System:A Review. Pharm. Nanotechnol..

[3] Bajracharya R., Song J.G., Back S.Y., Han H.-K. (2019). Recent Advancements in Non-Invasive Formulations for Protein Drug Delivery. Comput. Struct. Biotechnol. J..

[4] Peng H., Wang J., Chen J., Peng Y., Wang X., Chen Y., Kaplan D.L., Wang Q. (2023). Challenges and opportunities in delivering oral peptides and proteins. Expert Opin. Drug Deliv..

[5] Zhu Q., Chen Z., Paul P.K., Lu Y., Wu W., Qi J. (2021). Oral delivery of proteins and peptides: Challenges, status quo and future perspectives. Acta Pharm. Sin. B.

[6] Mehrotra S., Kalyan Bg P., Nayak P.G., Joseph A., Manikkath J. (2023). Recent progress in the oral delivery of therapeutic peptides and proteins: Overview of pharmaceutical strategies to overcome absorption hurdles. Adv. Pharm. Bull..

[7] Jacob S., Nair A.B., Boddu S.H.S., Gorain B., Sreeharsha N., Shah J. (2021). An Updated Overview of the Emerging Role of Patch and Film-Based Buccal Delivery Systems. Pharmaceutics.

[8] Montenegro-Nicolini M., Morales J.O. (2017). Overview and Future Potential of Buccal Mucoadhesive Films as Drug Delivery Systems for Biologics. Aaps PharmSciTech.

[9] Shipp L., Liu F., Kerai-Varsani L., Okwuosa T.C. (2022). Buccal films: A review of therapeutic opportunities, formulations & relevant evaluation approaches. J. Control Release.

[10] Ozon E.A., Sarbu I., Popovici V., Mitu M.A., Musuc A.M., Karampelas O., Velescu B.S. (2023). Three-Dimensional Printing Technologies in Oral Films Manufacturing—A Minireview. Processes.

[11] Fr S., Mullani A.K., V A.P.T., Koushik A., Chethan S., Alshehri S., Alsanie W.F., Alamri A.S., Alhomrani M., Alshammary A.F. (2025). Development and evaluation of mucoadhesive buccal films for sustained release of glipizide using natural proteins. Drug Dev. Ind. Pharm..

[12] Jovanović M., Petrović M., Cvijić S., Tomić N., Stojanović D., Ibrić S., Uskoković P. (2021). 3D Printed Buccal Films for Prolonged-Release of Propranolol Hydrochloride: Development, Characterization and Bioavailability Prediction. Pharmaceutics.

[13] Ebrahimi S.B., Samanta D. (2023). Engineering protein-based therapeutics through structural and chemical design. Nat. Commun..

[14] Chan A., Tsourkas A. (2024). Intracellular Protein Delivery: Approaches, Challenges, and Clinical Applications. BME Front..

[15] Dimitrov D.S., Voynov V., Caravella J.A. (2012). Therapeutic Proteins. Therapeutic Proteins.

[16] Akbarian M., Chen S.-H. (2022). Instability Challenges and Stabilization Strategies of Pharmaceutical Proteins. Pharmaceutics.

[17] Vaishya R., Khurana V., Patel S., Mitra A.K. (2015). Long-term delivery of protein therapeutics. Expert Opin. Drug Deliv..

[18] Rondon A., Mahri S., Morales-Yanez F., Dumoulin M., Vanbever R. (2021). Protein Engineering Strategies for Improved Pharmacokinetics. Adv. Funct. Mater..

[19] Zhu Y., Zhuang W., Cheng H. (2025). Strategies to Enhance Protein Delivery. Langmuir.

[20] Mehrdadi S. (2023). Lipid-based nanoparticles as oral drug delivery systems: Overcoming poor gastrointestinal absorption and enhancing bioavailability of peptide/protein-based drugs. Adv. Pharm. Bull..

[21] Masson P., Lushchekina S. (2022). Conformational Stability and Denaturation Processes of Proteins Investigated by Electrophoresis under Extreme Conditions. Molecules.

[22] Baral K.C., Choi K.Y. (2025). Barriers and Strategies for Oral Peptide and Protein Therapeutics Delivery: Update on Clinical Advances. Pharmaceutics.

[23] Tollinger M., Crowhurst K.A., Kay L.E., Forman-Kay J.D. (2003). Site-specific contributions to the pH dependence of protein stability. Proc. Natl. Acad. Sci. USA.

[24] Bischof J.C., He X. (2005). Thermal stability of proteins. Ann. N. Y. Acad. Sci..

[25] Krause M.E., Sahin E. (2019). Chemical and physical instabilities in manufacturing and storage of therapeutic proteins. Curr. Opin. Biotechnol..

[26] Li J., Krause M.E., Chen X., Cheng Y., Dai W., Hill J.J., Huang M., Jordan S., LaCasse D., Narhi L. (2019). Interfacial Stress in the Development of Biologics: Fundamental Understanding, Current Practice, and Future Perspective. AAPS J..

[27] Sarvepalli S., Pasika S.R., Verma V., Thumma A., Bolla S., Nukala P.K., Butreddy A., Bolla P.K. (2025). A Review on the Stability Challenges of Advanced Biologic Therapeutics. Pharmaceutics.

[28] Wang W., Roberts C.J. (2018). Protein aggregation—Mechanisms, detection, and control. Int. J. Pharm..

[29] Warne N., Mahler H.-C. (2018). Challenges in Protein Product Development.

[30] Shaji J., Patole V. (2008). Protein and Peptide drug delivery: Oral approaches. Indian J. Pharm. Sci..

[31] Carter P.J., Lazar G.A. (2018). Next generation antibody drugs: Pursuit of the “high-hanging fruit”. Nat. Rev. Drug Discov..

[32] Rispens T., Jiskoot W., Kijanka G., Crommelin D.J.A., Sindelar R.D., Meibohm B. (2024). Immunogenicity of Therapeutic Proteins. Pharmaceutical Biotechnology: Fundamentals and Applications.

[33] Jarvi N.L., Balu-Iyer S.V. (2021). Immunogenicity Challenges Associated with Subcutaneous Delivery of Therapeutic Proteins. BioDrugs.

[34] Binder U., Skerra A. (2025). Strategies for extending the half-life of biotherapeutics: Successes and complications. Expert Opin. Biol. Ther..

[35] Nguyen T.T.K., Pham K.-Y., Yook S. (2023). Engineered therapeutic proteins for sustained-release drug delivery systems. Acta Biomater..

[36] Van Witteloostuijn S.B., Pedersen S.L., Jensen K.J. (2016). Half-Life Extension of Biopharmaceuticals using Chemical Methods: Alternatives to PEGylation. ChemMedChem.

[37] Sánchez-Trasviña C., Flores-Gatica M., Enriquez-Ochoa D., Rito-Palomares M., Mayolo-Deloisa K. (2021). Purification of Modified Therapeutic Proteins Available on the Market: An Analysis of Chromatography-Based Strategies. Front. Bioeng. Biotechnol..

[38] Haddadzadegan S., Dorkoosh F., Bernkop-Schnürch A. (2022). Oral delivery of therapeutic peptides and proteins: Technology landscape of lipid-based nanocarriers. Adv. Drug Deliv. Rev..

[39] Dolas R., Kulkarni A., Gulecha V., Zalte A., Talele S., Bedarkar G. (2022). Buccal drug delivery system: A review. Int. J. Health Sci..

[40] Haju S., Yadav S., Baig R., Sawant G. (2021). Buccal Film: A Novel Approach for Oral Mucosal Drug Delivery System. Asian J. Pharm. Clin. Res..

[41] Chinna Reddy P., Chaitanya K.S.C., Madhusudan Rao Y. (2011). A review on bioadhesive buccal drug delivery systems: Current status of formulation and evaluation methods. Daru.

[42] Dhaifallah H. (2024). Review on Buccal Drug Delivery Systems. Int. J. Multidiscip. Res..

[43] Wanasathop A., Patel P.B., Choi H.A., Li S.K. (2021). Permeability of Buccal Mucosa. Pharmaceutics.

[44] Nair V.V., Cabrera P., Ramírez-Lecaros C., Jara M.O., Brayden D.J., Morales J.O. (2023). Buccal delivery of small molecules and biologics: Of mucoadhesive polymers, films, and nanoparticles—An update. Int. J. Pharm..

[45] Dubashynskaya N.V., Petrova V.A., Skorik Y.A. (2024). Biopolymer Drug Delivery Systems for Oromucosal Application: Recent Trends in Pharmaceutical R&D. Int. J. Mol. Sci..

[46] Southward J., Liu F., Aspinall S.R., Okwuosa T.C. (2025). Exploring the potential of mucoadhesive buccal films in geriatric medicine. Drug Dev. Ind. Pharm..

[47] AlMulhim F.M., Nair A.B., Aldhubiab B., Shah H., Shah J., Mewada V., Sreeharsha N., Jacob S. (2023). Design, Development, Evaluation, and In Vivo Performance of Buccal Films Embedded with Paliperidone-Loaded Nanostructured Lipid Carriers. Pharmaceutics.

[48] Carvalho F., Bruschi M., Evangelista R., Gremiao M. (2010). Mucoadhesive drug delivery systems. Braz. J. Pharm. Sci..

[49] Chede L.S., Wagner B.A., Buettner G.R., Donovan M.D. (2021). Electron Spin Resonance Evaluation of Buccal Membrane Fluidity Alterations by Sodium Caprylate and L-Menthol. Int. J. Mol. Sci..

[50] Caffarel-Salvador E., Kim S., Soares V., Tian R.Y., Stern S.R., Minahan D., Yona R., Lu X., Zakaria F.R., Collins J. (2021). A microneedle platform for buccal macromolecule delivery. Sci. Adv..

[51] Cheng H., Wang Y., Hong Y., Wu F., Shen L., Lin X. (2025). Characteristics, preparation and applicability in oral delivery systems of cellulose ether–based buccal films. Drug Deliv..

[52] Salih Z.T., Al-Mahmood A., Al-Mahmood S. (2023). Drug Delivery System Using a Buccal Film. Maaen J. Med. Sci..

[53] De Carvalho A.C.W., Paiva N.F., Demonari I.K., Duarte M.P.F., Do Couto R.O., De Freitas O., Vicentini F.T.M.D.C. (2023). The Potential of Films as Transmucosal Drug Delivery Systems. Pharmaceutics.

[54] Macedo A.S., Castro P.M., Roque L., Thomé N.G., Reis C.P., Pintado M.E., Fonte P. (2020). Novel and revisited approaches in nanoparticle systems for buccal drug delivery. J. Control Release.

[55] (2025). Aquestive Therapeutics Announces Positive Topline PK Results from Its Pediatric Study and Completes the NDA Submission for Anaphylm^TM^ (Epinephrine) Sublingual Film. https://investors.aquestive.com/news-releases/news-release-details/aquestive-therapeutics-announces-positive-topline-pk-results-its.

[56] Heng H.C., Rehman K., Zulfakar M.H. (2025). Molecular Docking-Guided Optimisation of an Aloe vera-Based Buccal Protein Delivery System. J. Sains Malays..

[57] Giovino C., Ayensu I., Tetteh J., Boateng J.S. (2013). An integrated buccal delivery system combining chitosan films impregnated with peptide loaded PEG-b-PLA nanoparticles. Colloids Surf. B Biointerfaces.

[58] Mortazavian E., Dorkoosh F.A., Rafiee-Tehrani M. (2014). Design, characterization and ex vivo evaluation of chitosan film integrating of insulin nanoparticles composed of thiolated chitosan derivative for buccal delivery of insulin. Drug Dev. Ind. Pharm..

[59] Diab M., Sallam A.-S., Hamdan I., Mansour R., Hussain R., Siligardi G., Qinna N., Khalil E. (2021). Characterization of Insulin Mucoadhesive Buccal Films: Spectroscopic Analysis and In Vivo Evaluation. Symmetry.

[60] Chamsai B., Opanasopit P., Samprasit W. (2023). Fast disintegrating dosage forms of mucoadhesive-based nanoparticles for oral insulin delivery: Optimization to in vivo evaluation. Int. J. Pharm..

[61] Peng H., Han B., Tong T., Jin X., Peng Y., Guo M., Li B., Ding J., Kong Q., Wang Q. (2024). 3D printing processes in precise drug delivery for personalized medicine. Biofabrication.

[62] Tian Y., Orlu M., Woerdenbag H.J., Scarpa M., Kiefer O., Kottke D., Sjöholm E., Öblom H., Sandler N., Hinrichs W.L.J. (2019). Oromucosal films: From patient centricity to production by printing techniques. Expert Opin. Drug Deliv..

[63] Takashima H., Tagami T., Kato S., Pae H., Ozeki T., Shibuya Y. (2022). Three-Dimensional Printing of an Apigenin-Loaded Mucoadhesive Film for Tailored Therapy to Oral Leukoplakia and the Chemopreventive Effect on a Rat Model of Oral Carcinogenesis. Pharmaceutics.

[64] Abdella S., Kim S., Afinjuomo F., Song Y., Upton R., Garg S. (2024). Combining the potential of 3D printed buccal films and nanostructured lipid carriers for personalised cannabidiol delivery. Drug Deliv. Transl. Res..

[65] Bhawale R., Suryavanshi P., Banerjee S. (2023). Three-dimensional (3D) printing of oral dental films (ODFs) using blended Compactcel^®^ polymers through semi-solid extrusion (SSE) bioprinter. Bioprinting.

[66] Brokmann F., Grzam S., Kandzi J.F., Krause J. (2025). 3D printed mucoadhesive films: Individualized drug dosing for localized drug delivery. Eur. J. Pharm. Sci..

[67] Chen Y., Mutukuri T.T., Wilson N.E., Zhou Q. (2021). (Tony) Pharmaceutical protein solids: Drying technology, solid-state characterization and stability. Adv. Drug Deliv. Rev..

[68] Development and Evaluation of 3D-Printed Losartan Potassium Tablets Using Semi-Solid Extrusion: The Effect of Geometry, Drug Loading and Superdisintegrant. https://www.mdpi.com/1424-8247/18/10/1504.

[69] Pardeshi S.R., Deshmukh N.S., Telange D.R., Nangare S.N., Sonar Y.Y., Lakade S.H., Harde M.T., Pardeshi C.V., Gholap A., Deshmukh P.K. (2023). Process development and quality attributes for the freeze-drying process in pharmaceuticals, biopharmaceuticals and nanomedicine delivery: A state-of-the-art review. Future J. Pharm. Sci..

[70] Azad M.A., Olawuni D., Kimbell G., Badruddoza A.Z.M., Hossain M.S., Sultana T. (2020). Polymers for Extrusion-Based 3D Printing of Pharmaceuticals: A Holistic Materials-Process Perspective. Pharmaceutics.

[71] Xu T., Li H., Xia Y., Ding S., Yang Q., Yang G. (2023). Three-Dimensional-Printed Oral Films Based on LCD: Influence Factors of the Film Printability and Received Qualities. Pharmaceutics.

[72] Abdella S., Afinjuomo F., Song Y., Upton R., Garg S. (2022). 3D printed bilayer mucoadhesive buccal film of estradiol: Impact of design on film properties, release kinetics and predicted in vivo performance. Int. J. Pharm..

[73] Eleftheriadis G.K., Ritzoulis C., Bouropoulos N., Tzetzis D., Andreadis D.A., Boetker J., Rantanen J., Fatouros D.G. (2019). Unidirectional drug release from 3D printed mucoadhesive buccal films using FDM technology: In vitro and ex vivo evaluation. Eur. J. Pharm. Biopharm..

[74] Saleh-Bey-Kinj Z., Heller Y., Socratous G., Christodoulou P. (2025). 3D Printing in Oral Drug Delivery: Technologies, Clinical Applications and Future Perspectives in Precision Medicine. Pharmaceuticals.

[75] Vaz V.M., Kumar L. (2021). 3D Printing as a Promising Tool in Personalized Medicine. AAPS PharmSciTech.

[76] Gupta M.S., Kumar T.P., Davidson R., Kuppu G.R., Pathak K., Gowda D.V. (2021). Printing Methods in the Production of Orodispersible Films. AAPS PharmSciTech.

[77] Buanz A.B.M., Belaunde C.C., Soutari N., Tuleu C., Gul M.O., Gaisford S. (2015). Ink-jet printing versus solvent casting to prepare oral films: Effect on mechanical properties and physical stability. Int. J. Pharm..

[78] Uddin M.J., Hassan J., Douroumis D. (2022). Thermal Inkjet Printing: Prospects and Applications in the Development of Medicine. Technologies.

[79] Zub K., Hoeppener S., Schubert U.S. (2022). Inkjet Printing and 3D Printing Strategies for Biosensing, Analytical, and Diagnostic Applications. Adv. Mater..

[80] Foo W.C., Widjaja E., Khong Y.M., Gokhale R., Chan S.Y. (2018). Application of miniaturized near-infrared spectroscopy for quality control of extemporaneous orodispersible films. J. Pharm. Biomed. Anal..

[81] Konta A.A., García-Piña M., Serrano D.R. (2017). Personalised 3D Printed Medicines: Which Techniques and Polymers Are More Successful?. Bioengineering.

[82] Melocchi A., Uboldi M., Cerea M., Foppoli A., Maroni A., Moutaharrik S., Palugan L., Zema L., Gazzaniga A. (2020). A Graphical Review on the Escalation of Fused Deposition Modeling (FDM) 3D Printing in the Pharmaceutical Field. J. Pharm. Sci..

[83] Wickramasinghe S., Do T., Tran P. (2020). FDM-Based 3D Printing of Polymer and Associated Composite: A Review on Mechanical Properties, Defects and Treatments. Polymers.

[84] Mazzanti V., Malagutti L., Mollica F. (2019). FDM 3D Printing of Polymers Containing Natural Fillers: A Review of their Mechanical Properties. Polymers.

[85] Arief R.K., Adesta E., Hilmy I. (2019). Hardware improvement of FDM 3D printer: Issue of bed leveling failures. Int. J. Innov. Technol. Explor. Eng..

[86] Khalid G.M., Billa N. (2022). Solid Dispersion Formulations by FDM 3D Printing-A Review. Pharmaceutics.

[87] Annaji M., Ramesh S., Poudel I., Govindarajulu M., Arnold R.D., Dhanasekaran M., Babu R.J. (2020). Application of Extrusion-Based 3D Printed Dosage Forms in the Treatment of Chronic Diseases. J. Pharm. Sci..

[88] Long J., Gholizadeh H., Lu J., Bunt C., Seyfoddin A. (2017). Application of Fused Deposition Modelling (FDM) Method of 3D Printing in Drug Delivery. Curr. Pharm. Des..

[89] Seoane-Viaño I., Januskaite P., Alvarez-Lorenzo C., Basit A.W., Goyanes A. (2021). Semi-solid extrusion 3D printing in drug delivery and biomedicine: Personalised solutions for healthcare challenges. J. Control Release.

[90] Cano-Vicent A., Tambuwala M., Hassan S.S., Barh D., Aljabali A., Birkett M., Arjunan A., Serrano-Aroca Á. (2021). Fused Deposition Modelling: Current Status, Methodology, Applications and Future Prospects. Addit. Manuf..

[91] Borges A.F., Silva C., Coelho J.F.J., Simões S. (2015). Oral films: Current status and future perspectives: I—Galenical development and quality attributes. J. Control Release.

[92] Madžarević M., Ibrić S. (2021). Evaluation of exposure time and visible light irradiation in LCD 3D printing of ibuprofen extended release tablets. Eur. J. Pharm. Sci..

[93] Quan H., Zhang T., Xu H., Luo S., Nie J., Zhu X. (2020). Photo-curing 3D printing technique and its challenges. Bioact. Mater..

[94] Shahrubudin N., Lee T.C., Ramlan R. (2019). An Overview on 3D Printing Technology: Technological, Materials, and Applications. Proceedings of the 2nd International Conference on Sustainable Materials Processing and Manufacturing, Sun City, South Africa, 8–10 March 2019.

[95] Al Mousawi A., Poriel C., Dumur F., Toufaily J., Hamieh T., Fouassier J., Lalevée J. (2017). Zinc Tetraphenylporphyrin as High Performance Visible Light Photoinitiator of Cationic Photosensitive Resins for LED Projector 3D Printing Applications. Macromolecules.

[96] Montenegro-Nicolini M., Miranda V., Morales J.O. (2017). Inkjet Printing of Proteins: An Experimental Approach. AAPS J..

[97] Montenegro-Nicolini M., Reyes P.E., Jara M.O., Vuddanda P.R., Neira-Carrillo A., Butto N., Velaga S., Morales J.O. (2018). The Effect of Inkjet Printing over Polymeric Films as Potential Buccal Biologics Delivery Systems. AAPS PharmSciTech.

[98] Alzhrani R.F., Xu H., Zhang Y., Maniruzzaman M., Cui Z. (2024). Development of novel 3D printable inks for protein delivery. Int. J. Pharm..

[99] Zhang Y., Dong L., Wang K., Cheng Y., Gao T., Yang J., Shen X., Cao Y., Xue B. (2025). Protein-based double-network hydrogels mimicking oral mucosa. Front. Chem..

[100] Aran K., Chooljian M., Paredes J., Rafi M., Lee K., Kim A.Y., An J., Yau J.F., Chum H., Conboy I. (2017). An oral microjet vaccination system elicits antibody production in rabbits. Sci. Transl. Med..

[101] Monou P.K., Andriotis E.G., Tsongas K., Tzimtzimis E.K., Katsamenis O.L., Tzetzis D., Anastasiadou P., Ritzoulis C., Vizirianakis I.S., Andreadis D. (2023). Fabrication of 3D Printed Hollow Microneedles by Digital Light Processing for the Buccal Delivery of Actives. ACS Biomater. Sci. Eng..

[102] Shah S., Patel P., Ferguson A., Bagwe P., Kale A., Adediran E., Singh R., Arte T., Pasupuleti D., Uddin M.N. (2024). Buccal Administration of a Zika Virus Vaccine Utilizing 3D-Printed Oral Dissolving Films in a Mouse Model. Vaccines.

[103] Kayadurmus H.M., Rezaei A., Ilhan E., Cesur S., Sahin A., Gunduz O., Kalaskar D.M., Ekren N. (2024). Whey protein-loaded 3D-printed poly (lactic) acid scaffolds for wound dressing applications. Biomed. Mater..

[104] Barekat S., Ubeyitogullari A. (2025). Developing hydrophobic-hydrophilic protein structures by 3D food printing of sorghum and soy protein gels. J. Food Eng..

[105] Barekat S., Ubeyitogullari A. (2025). Maximizing sorghum proteins printability: Optimizing gel formulation and 3D-printing parameters to develop a novel bioink. Int. J. Biol. Macromol..

[106] Samiei N. (2020). Recent trends on applications of 3D printing technology on the design and manufacture of pharmaceutical oral formulation: A mini review. Beni-Suef Univ. J. Basic. Appl. Sci..

[107] Jacob S., Boddu S.H.S., Bhandare R., Ahmad S.S., Nair A.B. (2023). Orodispersible Films: Current Innovations and Emerging Trends. Pharmaceutics.

[108] Mancilla-De-la-Cruz J., Rodriguez-Salvador M., An J., Chua C.K. (2022). Three-Dimensional Printing Technologies for Drug Delivery Applications: Processes, Materials, and Effects. Int. J. Bioprinting.

